# Psychological Comorbidities Among Patients With Type 2 Diabetes Mellitus (T2DM) in Anakaputhur, Chennai: A Cross-Sectional Study

**DOI:** 10.7759/cureus.91783

**Published:** 2025-09-07

**Authors:** Praveen Murugesan, Anantha Eashwar V M, Angeline Grace, Aljin V

**Affiliations:** 1 Community Medicine, Sree Balaji Medical College and Hospital, Chennai, IND

**Keywords:** anxiety, chennai, depression, diabetes mellitus, psychological stress

## Abstract

Background

Psychological comorbidities such as depression, anxiety, and stress may occur in patients with type 2 diabetes mellitus (T2DM), potentially influencing clinical outcomes. Data from semi-urban areas of India remain limited, and differences in healthcare availability and social factors may influence these outcomes. This study aims to assess the prevalence and socio-demographic determinants of depression, anxiety, and stress in individuals with T2DM and to explore the interrelationships among these comorbidities.

Methodology

An analytical cross-sectional study was conducted over three months in the community, served by the Urban Health Training Centre (UHTC) of Sree Balaji Medical College and Hospital in Anakaputhur, Chennai, India. The study population included adults (≥18 years) diagnosed with T2DM and receiving treatment for at least six months. Individuals with pre-existing psychiatric disorders or advanced diabetic complications were excluded. A multistage sampling strategy was applied: in the first stage, eligible patients were identified from outpatient medical records at the UHTC; in the second stage, a simple random sampling method was used to select 412 participants from this list. Data were collected through household visits, where trained investigators administered a structured questionnaire to obtain socio-demographic information, clinical history, and psychological distress levels using the Depression Anxiety Stress Scale-21 (DASS-21). Data were entered in Microsoft Excel (Microsoft Corp., Redmond, WA, USA) and analyzed using IBM SPSS Statistics for Windows, V. 25.0 (IBM Corp., Armonk, NY, USA). Descriptive statistics were used to determine prevalence rates, while the chi-squared test assessed associations between psychological comorbidities and socio-demographic variables. Multivariate logistic regression was performed to identify independent predictors, with results expressed as odds ratios (OR) and 95% confidence intervals (CI).

Results

Psychological comorbidities were prevalent among participants: 44.7% (n=184) experienced stress, 51.5% (n=212) had anxiety, and 48.8% (n=201) suffered from depression. A significant overlap existed among these conditions, with 74.1% of those with stress also experiencing anxiety and 82.1% of depressed individuals reporting anxiety (p<0.001). Significant socio-demographic predictors included older age (51-60 years), which was associated with higher odds of stress (OR=3.68; p=0.004), anxiety (OR=3.54; p=0.007), and depression (OR=3.92; p=0.004). Female participants had a higher likelihood of experiencing stress (adjusted OR=1.36; 95% CI: 0.76-2.43; p=0.3) and were independently more likely to have anxiety (OR=2.85; p<0.001). Additionally, student status emerged as a strong predictor of depression (OR=8.19; p<0.001).

Conclusion

Psychological comorbidities were common among individuals with T2DM in this peri-urban Indian setting. Higher prevalence was observed in older adults, women, and those identified as students. Considerable overlap existed between depression, anxiety, and stress. These findings underscore the importance of incorporating routine mental health screening into diabetes care and implementing targeted interventions for vulnerable groups to improve their overall psychological well-being.

## Introduction

In urban India, type 2 diabetes mellitus (T2DM) is a major public health concern, with prevalence rates ranging from 8% to 15% in adults [1]. Psychological comorbidities, including stress, anxiety, and depression, are often linked to T2DM and can result in higher morbidity, early mortality, and a lower quality of life. T2DM affects an estimated 537 million adults globally, with over 77 million cases reported in India in 2019. This number is predicted to rise to 134 million by 2045, and mental health issues are commonly underdiagnosed and undertreated in this population [2]. In India, the combined prevalence of stress, anxiety, and depression was between 44% and 70% of T2DM patients, which is much higher than the general population. These mental health disorders impair glycemic management, lower adherence to self-care practices, and raise the risk of microvascular and macrovascular issues [3]. While global estimates are typically between 30% and 40%, Indian research indicates that the prevalence of depression alone varies widely, with figures ranging from as low as 7% to as high as 84% [4].

Stress and anxiety have a similar impact on the burden of disease, despite the fact that there is a dearth of local data from Chennai regarding the combined prevalence of these psychological disorders [5]. In South Asia, depression rates among T2DM patients range from 8.5% to 32.5%, with higher rates observed in women, insulin users, and those with concurrent conditions from diabetes [6]. Although the International Diabetes Federation recommends routine mental health evaluations for diabetics, thorough psychiatric evaluations are rarely carried out in South India [7], and the psychosocial components of therapy are frequently neglected [8]. Poor treatment adherence and a reduction in health-related quality of life are the outcomes of this oversight [9].

Furthermore, compared to people without diabetes, T2DM patients who experience melancholy have substantially higher healthcare utilization and expenses, according to international studies [10]. The urgent demand for integrated screening and management strategies is highlighted by the co-occurrence of non-communicable diseases and common mental health conditions such as stress, anxiety, and depression [11]. Glycemic management and overall disease outcomes might be significantly hampered by behavioral and psychosocial variables [12]. Therefore, the purpose of this study is to determine the prevalence and demographic predictors of depression, anxiety, and stress among patients with T2DM and to examine the interrelationship between these psychological comorbidities within this population.

## Materials and methods

Study setting 

Adults with T2DM residing in the Anakaputhur service area and registered with the Urban Health Training Centre (UHTC) of Sree Balaji Medical College and Hospital, Chennai, India, were recruited for an analytical cross-sectional study after obtaining approval from the institute's Institutional Human Ethics Committee (approval number: 002/SBMCH/IHEC/2025/2392). The sampling frame was the UHTC clinic registry, and data were collected through household visits, representing a clinic-based sample with community-level data collection. Anakaputhur was chosen because of its diverse peri-urban population, accessibility, practicality, and lack of adequate representation in the body of current literature. The total duration of the study was three months, from April 1, 2025, to June 30, 2025, with data collection beginning on April 3, 2025, and concluding on June 28, 2025..

Inclusion and exclusion criteria 

Eligible adults were those who were 18 years of age or older, had a verified diagnosis of T2DM, and had been receiving treatment for at least six months. Participants were included if they could speak in Tamil or English and had finished the questionnaire completely. To be sure that the psychological outcomes examined were directly related to T2DM, those with severe acute diabetic complications that required hospitalization, pre-existing psychiatric problems, or gestational diabetes were excluded.

Sample size calculation 

The sample size was determined based on a previously noted prevalence of depression at 49.2% among patients with T2DM, as stated by Kant et al. [4]. Utilizing the formula n=Z^2^ pq/L^2^ with Z=1.96 (for a 95% confidence level (CI)), p=49.2%, q=50.8%, and L=5% allowable error, the calculated sample size needed was 384. By accounting for a 5% non-response rate, the target was raised to 403. Ultimately, complete data were collected from 412 participants, surpassing the required minimum sample size.

Sampling technique 

The study employed a multistage random sampling technique. Clinic records were used to build a line list of T2DM patients who were registered at the UHTC. Patients between the ages of 18 and 65 were identified, and participants were chosen by simple random sampling using a lottery approach. Two follow-up visits were scheduled on different days if a selected individual was unavailable for the initial home visit; if the individual was still unavailable, the next eligible patient on the list was added.

Data collection tool 

Trained postgraduate residents from the Department of Community Medicine conducted in-person home interviews to collect data. Clinical history, lifestyle variables, and socio-demographic data were gathered using a pre-tested standardized questionnaire (see Appendices). Psychological comorbidities were assessed using the Depression Anxiety Stress Scale-21 (DASS-21), a validated self-report instrument developed by Lovibond and Lovibond [13]. The scale consists of 21 items across three subscales, depression, anxiety, and stress, with higher scores indicating greater symptom severity. Throughout every contact, participants' comfort, privacy, and ethical interviewing approaches were given first priority.

Data analysis 

Microsoft Excel (Microsoft Corp., Redmond, WA, USA) was used to record the data, and IBM SPSS Statistics for Windows, V. 25.0 (IBM Corp., Armonk, NY, USA), was used for analysis. The prevalence of stress, anxiety, and depression were assessed using descriptive statistics. To investigate relationships between independent factors and psychological comorbidities, the chi-squared test was utilized. In order to find independent factors, multivariate logistic regression was used, and odds ratios (OR) and 95% CI were reported. Statistical significance was defined as a p-value of less than 0.05. 

## Results

Socio-demographic and clinical profile of participants

Although no standalone table was created for socio-demographic and clinical characteristics, these variables are incorporated within the univariate and multivariate analyses for stress, anxiety, and depression. The study population comprised 412 patients with T2DM, with a mean age of 42.3 years (SD±12.5). Females constituted two-thirds of the sample (66.5%), and the most represented age groups were 31-50 years (39.3%) and 51-60 years (36.9%). More than half of the participants (56.3%) had attained a college-level education or higher, and the majority (68.0%) were married. Overall, the sample reflects a predominantly middle-aged, female, and relatively well-educated population, with a substantial proportion having an established history of T2DM.

Table 1 presents univariate and multivariate analyses of demographic factors associated with stress. In univariate analysis, stress prevalence increased significantly with age (p<0.001), peaking in those aged 51-60 years (28.3%). Males, single individuals, those from low-income or unemployed groups, nuclear families, and those with a family history of diabetes or frequent alcohol use also showed higher stress levels. After adjustment, age remained the strongest predictor, with individuals >60 years (OR=4.12; p=0.002), 51-60 years (OR=3.68; p=0.004), and 41-50 years (OR=2.45; p=0.047) having significantly higher odds compared to the 18-30-year age group. Female participants had a significantly higher likelihood of experiencing stress (adjusted OR=1.36; 95% CI: 0.76-2.43; p=0.3) with a prevalence of 61.4%, indicating potential gender-related stressors such as caregiving responsibilities or societal expectations.

**Table 1:** Univariate and multivariate analyses of demographic variables associated with stress in patients with type 2 diabetes mellitus Univariate analysis was conducted using the chi-squared test and multivariate analysis using logistic regression (enter method). *p-value from univariate analysis (chi-squared test), significant at p<0.05; **p-value from logistic regression analysis, significant at p<0.05; ^95% CI: 95% confidence interval; OR: odds ratio Scores derived using the Depression Anxiety Stress Scale-21 (DASS-21) [13].

Variable	Stress				P-value*	Adjusted OR (95% CI)^	P-value**
	Stressed (n=184)		Normal (n=228)				
	n	%	N	%			
Age (in years)							
18-30	30	16.3	151	66.2	<0.001	Ref	-
31-40	35	19	47	20.6		1.89 (0.78-4.56)	0.162
41-50	50	27.2	12	5.3		2.45 (1.02-5.87)	0.047
51-60	52	28.3	6	2.6		3.68 (1.52-8.92)	0.004
>60	17	9.2	12	5.3		4.12 (1.67-10.18)	0.002
Gender							
Male	71	38.59	65	28.51	0.031	Ref	-
Female	113	61.41	163	71.49		1.36 (0.76-2.43)	0.3
Marital status							
Married	106	57.61	165	73.33	0.002	Ref	-
Unmarried	62	33.7	52	23.11		1.794 (0.804-4.001)	0.153
Divorced	12	6.52	8	3.56		2.477 (0.659-9.310)	0.179
Widowed	4	2.17	0	0		0.000 (0.000-0.000)	0.072
Education status							
Uneducation	16	8.7	4	1.75	0.024	Ref	-
Primary	19	10.33	25	10.96		0.938 (0.313-2.811)	0.909
Secondary and higher secondary	46	25	65	28.51		1.712 (0.149-19.671)	0.666
Graduate	79	42.93	108	47.37		2.187 (0.855-5.597)	0.103
Postgraduate	24	13.04	26	11.4		2.822 (0.709-11.230)	0.14
Employment							
Unemployed	21	11.41	14	6.14	<0.001	Ref	-
Employed	66	35.87	45	19.74		0.938 (0.313-2.811)	0.909
Retired	7	3.8	3	1.32		1.712 (0.149-19.671)	0.666
Student	79	42.93	154	67.54		2.187 (0.855-5.597)	0.103
Homemaker	11	5.98	12	5.26		2.822 (0.709-11.230)	0.14
Monthly income							
<10000	38	20.65	66	28.95	0.043	1.181 (0.547-2.551)	0.672
10000-24999	42	22.83	65	28.51		0.750 (0.348-1.616)	0.461
25000-50000	55	29.89	50	21.93		0.512 (0.249-1.052)	0.068
>50000	49	26.63	47	20.61		Ref	-
Type of family							
Nuclear	91	49.46	144	63.16	0.008	Ref	-
Joint	88	47.83	75	32.89		0.878 (0.531-1.450)	0.61
Extended	5	2.72	9	3.95		6.962 (0.799-60.596)	0.079
Place of residence							
Urban	127	69.02	170	74.56	0.213	-	-
Rural	57	30.98	58	25.44		-	-
Family history							
Yes	55	29.89	26	11.4	<0.001	0.510 (0.258-1.008)	0.053
No	129	70.11	202	88.6		Ref	-
How often have you consumed alcohol in the past 12 months?							
Daily or almost daily	22	11.96	3	1.32	<0.001	0.121 (0.030-0.483)	0.003
1-3 times a month	44	23.91	13	5.7		0.131 (0.058-0.295)	<0.001
Less than once a month	22	11.96	14	6.14		0.327 (0.133-0.802)	0.015
No	96	52.17	198	86.84		Ref	-

Table 2 shows the univariate and multivariate analyses for anxiety. Anxiety prevalence was highest in the 51-60-year age group (25.9%; adjusted OR=3.54; 95% CI: 1.41-8.87; p=0.007). Females had a markedly higher prevalence of 59% vs 41% in males, and female sex remained an independent predictor (OR=2.85; 95% CI: 1.59-5.09; p<0.001). In multivariate analysis, the 51-60-year age group retained a significant association (OR=3.54; p=0.007). 

**Table 2:** Univariate and multivariate analyses of demographic variables associated with anxiety in patients with type 2 diabetes mellitus Univariate analysis was conducted using the chi-squared test and multivariate analysis using logistic regression (enter method). *p-value from univariate analysis (chi-squared test), significant at p<0.05; **p-value from logistic regression analysis, significant at p<0.05; ^95% CI: 95% confidence interval; OR: odds ratio Scores derived using the Depression Anxiety Stress Scale-21 (DASS-21) [13].

Variable	Anxiety				P-value*	Adjusted OR (95% CI)^	P-value**
	Anxiety (n=212)		Normal (n=200)				
	n	%	n	%			
Age (in years)							
18-30	50	23.6	131	65.5	<0.001	Ref	-
31-40	38	17.9	44	22		1.45 (0.68-3.10)	0.341
41-50	50	23.6	12	6		2.78 (1.89-3.14)	0.002
51-60	55	25.9	3	1.5		3.54 (1.41-8.87)	0.007
>60	19	9	10	5		1.12 (0.89-5.04)	0.214
Gender							
Male	87	41.04	49	24.5	<0.001	Ref	-
Female	125	58.96	151	75.5		2.853 (1.596-5.098)	<0.001
Marital status							
Married	123	58.02	148	75.13	0.001	Ref	-
Unmarried	72	33.96	42	21.32		0.774 (0.346-1.729)	0.532
Divorced	13	6.13	7	3.55		1.458 (0.366-5.814)	0.593
Widowed	4	1.89	0	0		0.000 (0.000-0.000)	0.999
Education status							
Uneducated	17	8.02	3	1.5	0.001	Ref	-
Primary	30	14.15	14	7		2.108 (0.299-14.860)	0.454
Secondary and higher secondary	47	22.17	64	32		3.633 (0.554-23.819)	0.179
Graduate	95	44.81	92	46		3.054 (0.502-18.579)	0.226
Postgraduate	23	10.85	27	13.5		6.883 (1.059-44.734)	0.043
Employment							
Unemployed	26	12.26	9	4.5	0.001	Ref	-
Employed	66	31.13	45	22.5		2.616 (0.820-8.343)	0.104
Retired	7	3.3	3	1.5		3.794 (0.359-40.085)	0.268
Student	97	45.75	136	68		5.285 (1.889-14.786)	0.002
Homemaker	16	7.55	7	3.5		2.718 (0.608-12.158)	0.191
Monthly income							
<10000	49	23.11	55	27.5	0.044	0.610 (0.288-1.291)	0.196
10000-24999	48	22.64	59	29.5		0.604 (0.285-1.278)	0.187
25000-50000	66	31.13	39	19.5		0.386 (0.189-0.786)	0.009
>50000	49	23.11	47	23.5		Ref	-
Type of family							
Nuclear	117	55.19	118	59	0.516	Ref	-
Joint	89	41.98	74	37		1.628 (0.977-2.712)	0.061
Extended	6	2.83	8	4		8.297 (1.237-55.635)	0.029
Place of residence							
Urban	148	69.81	149	74.5	0.289	-	-
Rural	64	30.19	51	25.5		-	-
Family history							
Yes	57	26.89	24	12	<0.001	0.573 (0.290-1.133)	0.109
No	155	73.11	176	88		Ref	-
How often have you consumed alcohol in the past 12 months?							
Daily or almost daily	22	10.38	3	1.5	<0.001	0.176 (0.045-0.688)	0.012
1-3 times a month	46	21.7	11	5.5		0.220 (0.099-0.491)	<0.001
Less than once a month	23	10.85	13	6.5		0.519 (0.207-1.300)	0.161
No	121	57.08	173	86.5		Ref	-

Table 3 details the univariate and multivariate associations between demographic variables and depression. Depression prevalence was highest in the 51-60-year age group (24.9%), with age showing a strong association (p<0.001). After adjustment, individuals aged 51-60 years had almost fourfold higher odds of depression (OR=3.92; p=0.004) compared with the youngest group. In the multivariable analysis, being a student was strongly associated with depression (adjusted OR=8.19; 95% CI: 3.06-21.94; p<0.001), suggesting that academic workload and urban lifestyle challenges may contribute substantially to this elevated risk.

**Table 3:** Univariate and multivariate analyses of demographic variables associated with depression in patients with type 2 diabetes mellitus Univariate analysis was conducted using the chi-squared test and multivariate analysis using logistic regression (enter method). *p-value from univariate analysis (chi-squared test), significant at p<0.05; **p-value from logistic regression analysis, significant at p<0.05; ^95% CI: 95% confidence interval; OR: odds ratio Scores derived using the Depression Anxiety Stress Scale-21 (DASS-21) [13].

Variable	Depression				P-value*	Adjusted OR (95% CI)^	P-value**
	Depressed (n=201)		Normal (n=211)				
	n	%	n	%			
Age (in years)							
18-30	50	24.9	131	62.1	<0.001	Ref	-
31-40	40	19.9	42	19.9		1.17 (1.06-2.21)	0.081
41-50	44	21.9	18	8.5		2.10 (0.87-5.08)	0.092
51-60	50	24.9	8	3.8		3.92 (1.52-8.92)	0.004
>60	17	8.5	12	5.7		1.25 (0.50-3.10)	0.62
Gender							
Male	70	34.83	66	31.28	0.444	-	-
Female	131	65.17	145	68.72		-	-
Marital status							
Married	113	57.07	158	74.88	0.001	Ref	-
Unmarried	69	34.85	45	21.33		1.291 (0.590-2.824)	0.522
Divorced	12	6.06	8	3.79		2.198 (0.607-7.962)	0.23
Widowed	4	2.02	0	0		0.000 (0.00,0.00)	0.999
Education status							
Uneducated	15	7.46	5	2.37	0.003	Ref	-
Primary	27	13.43	17	8.06		0.947 (0.222-4.048)	0.942
Secondary and higher secondary	40	19.9	71	33.65		1.766 (0.446-6.989)	0.418
Graduate	94	46.77	93	44.08		1.098 (0.296-4.074)	0.889
Postgraduate	25	12.44	25	11.85		1.979 (0.473-8.277)	0.35
Employment							
Unemployed	28	13.93	7	3.32	<0.001	Ref	-
Employed	69	34.33	42	19.91		3.566 (1.167-10.901)	0.026
Retired	7	3.48	3	1.42		5.395 (0.648-44.946)	0.119
Student	83	41.29	150	71.09		8.190 (3.057-21.940)	<0.001
Homemaker	14	6.97	9	4.27		6.321 (1.505-26.547)	0.012
Monthly income							
<10000	53	26.37	51	24.17	0.139	-	-
10000-24999	42	20.9	65	30.81		-	-
25000-50000	54	26.87	51	24.17		-	-
>50000	52	25.87	44	20.85		-	-
Type of family							
Nuclear	108	53.73	127	60.19	0.313	-	-
Joint	87	43.28	76	36.02		-	-
Extended	6	2.99	8	3.79		-	-
Place of residence							
Urban	143	71.14	154	72.99	0.677	-	-
Rural	58	28.86	57	27.01		-	-
Family history							
Yes	54	26.87	27	12.8	<0.001	0.785 (0.407-1.512)	0.469
No	147	73.13	184	87.2		Ref	-
How often have you consumed alcohol in the past 12 months?							
Daily or almost daily	20	9.95	5	2.37	<0.001	0.229 (0.076-0.693)	0.009
1-3 times a month	49	24.38	8	3.79		0.100 (0.042-0.234)	<0.001
Less than once a month	24	11.94	12	5.69		0.316 (0.136-0.736)	0.008
No	108	53.73	186	88.15		Ref	-

**Figure 1:**
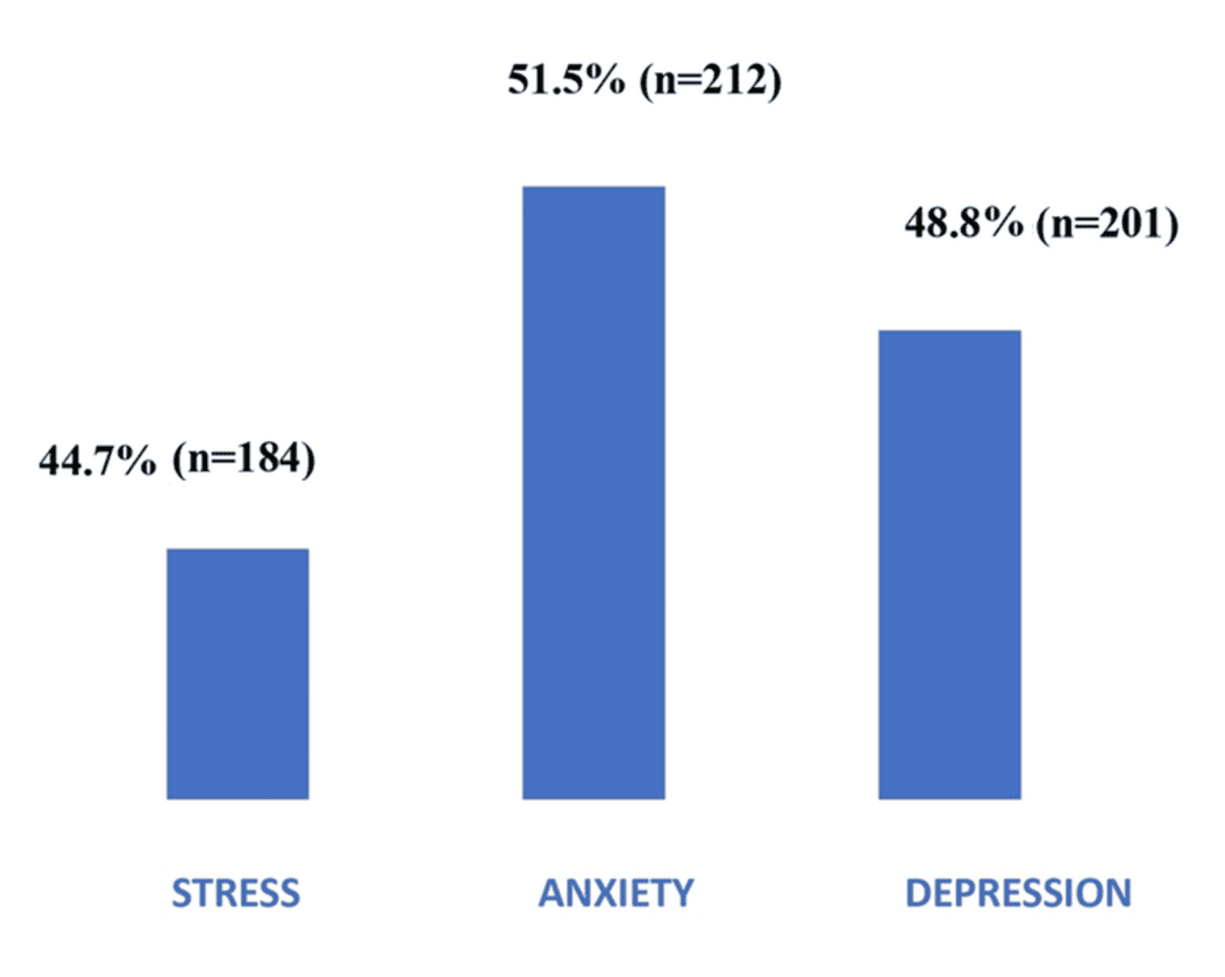
Distribution of stress, anxiety, and depression among the respondents Scores derived using the Depression Anxiety Stress Scale-21 (DASS-21) [13].

Figure 1 illustrates the distribution of stress, anxiety, and depression among the study participants. The prevalence of anxiety (51.5%; n=212), depression (48.8%; n=201), and stress (44.7%; n=184) was notably high. These rates exceed the reported global estimates of 20-40% for psychological distress in T2DM patients, underscoring a substantial mental health burden in this semi-urban South Indian population.

Table 4 illustrates the overlap between stress and anxiety, showing that 74.1% of stressed patients also experienced anxiety compared to 25.9% among those without stress. This difference was statistically significant (p<0.001), indicating a strong positive association. These results highlight the need for concurrent screening for anxiety in stressed T2DM patients.

**Table 4:** Association between stress and anxiety in patients with type 2 diabetes mellitus In this table, the association between stress and anxiety was examined using the chi-squared test of independence (χ² test). The results show a statistically significant association between stress and anxiety (χ²=152.698; p<0.001). *p-value from the chi-squared test, significant at p<0.05; χ²: chi-squared statistic Scores derived using the Depression Anxiety Stress Scale-21 (DASS-21) [13].

Variables	Anxiety	Normal	Total	χ²	P-value*
Stressed	157 (74.1)	27 (13.5)	184	152.698	<0.001
Normal	55 (25.9)	173 (86.5)	228		
Total	212	200	412		

Table 5 demonstrates a marked co-occurrence of depression and anxiety, with 82.1% of those with depression also reporting anxiety, compared to only 17.9% in non-depressed individuals. This statistically significant difference (p<0.001) reflects a strong positive association.

**Table 5:** Association between depression and anxiety in patients with type 2 diabetes mellitus In this table, the association between depression and anxiety was assessed using the chi-squared test of independence (χ² test). A statistically significant association was observed (χ²=193.697; p<0.001). *p-value from the chi-squared test, significant at p<0.05; χ²: chi-squared statistic Scores derived using the Depression Anxiety Stress Scale-21 (DASS-21) [13].

Variables	Anxiety	Normal	Total	χ²	P-value*
Depressed	174 (82.1)	27 (13.5)	201	193.697	<0.001
Normal	38 (17.9)	173 (86.5)	211		
Total	212	200	412		

## Discussion

In our cross-sectional study involving 412 individuals with T2DM in Chennai, we observed a substantial prevalence of psychological comorbidities: stress in 44.7% of participants, anxiety in 51.5%, and depression in 48.8%. These results align with the meta-analysis by Anderson et al. [14], which synthesized findings from 42 studies and reported that people with diabetes were about twice as likely to experience depression compared to those without the condition (OR=2.0; 95% CI: 1.8-2.2). Their analysis also highlighted a higher prevalence of depression in clinical populations versus community samples and among women (28%) compared to men (18%).

Kanwar et al. [15] reported a high burden of psychiatric morbidity among T2DM patients in a hilly region of North India, a finding that aligns with our results and underscores the need for integrating mental health evaluation into routine diabetes care. Similarly, Majeed et al. [16] observed that anxiety and depression are common in rural North Indian populations, with strong associations to poor glycemic control and longer disease duration. In Eastern India, Karpha et al. [17] further noted that such psychiatric conditions were significantly influenced by factors including low socioeconomic status, female gender, and the presence of diabetes-related comorbidities.

The prevalence observed in our cohort is in line with the findings of Surya et al. [18], who reported that individuals with diabetes in Kancheepuram district experienced considerable psychological distress and anxiety influenced by lifestyle and demographic factors. Likewise, in a tertiary care setting in South India, Dogra et al. [19] identified a high burden of diabetes-related distress and depression, particularly among patients with suboptimal metabolic control.

Particular attention should be paid to the link between depressive symptoms and adherence to self-care practices. Even mild to moderate depression can adversely influence disease outcomes, as demonstrated by Gonzalez et al. [20], who reported a clear inverse relationship between depression severity and compliance with medication and lifestyle recommendations. Supporting this, Mendenhall et al. [21], in a qualitative study from India, described how the bidirectional relationship between diabetes and depression is intensified by social stigma, psychological stressors, and the limited integration of mental health services into routine diabetes care.

Among Indian patients with T2DM, Sinha et al. [22] reported a strong association between poor glycemic control and diabetes-related distress and anxiety, which frequently overlaps with depressive symptoms. Similar observations were made by Kashif and Stanly [23], who identified female gender and pre-existing health conditions as significant predictors of depression in rural populations. Psychosocial factors also play a crucial role in adherence patterns; Rana et al. [24] underscored the importance of incorporating behavioral interventions into diabetes care, noting that feelings of disappointment, self-efficacy, and perceived disease severity are major determinants of self-care practices. Further supporting this, Schram et al. [25], in a study from the European Depression in Diabetes (EDID) group, demonstrated that depression not only diminishes quality of life but also increases the risk of complications and mortality in this patient group.

Tripathi et al. [26] identified female gender, obesity, unmarried status, poor glycemic control, and the presence of comorbidities as key risk factors for depression and anxiety, affecting 20.3% and 17.3% of Indian patients with T2DM, respectively. Additionally, longitudinal data from Lin et al. [27] demonstrated that stress and depression independently increased the risk of both microvascular and macrovascular complications, even after adjusting for self-care behaviors and other clinical variables.

Taken together, findings from our study and previous literature highlight the pressing need for routine mental health assessment, incorporation of psychosocial support, and tailored, patient-centered approaches in the management of T2DM. Early identification and treatment of depression, anxiety, and stress have the potential to improve psychological outcomes, optimize glycemic control, and lower the long-term risk of diabetes-related complications.

Limitations

The generalizability of this study is limited, as it was conducted in a single peri-urban outpatient clinic and may not fully reflect patterns seen in rural settings or densely populated urban areas. Owing to its cross-sectional design, causal relationships between psychological conditions and diabetes-related factors cannot be established. Self-reported information on alcohol consumption and lifestyle behaviors may also be influenced by recall bias or social desirability. Furthermore, while the DASS-21 serves as a valid screening instrument, it does not replace a formal psychiatric assessment, and no definitive clinical diagnoses were made in this study. In addition, restricting participation to Tamil- and English-speaking individuals and including only complete responses may have introduced selection bias. In our study, 18 otherwise eligible participants were excluded due to language barriers and 11 due to incomplete questionnaires.

Recommendation

Routine screening for stress, anxiety, and depression should be incorporated into diabetes management at all levels, with special focus on resource-limited settings. Healthcare providers should receive training to promptly recognize and refer patients experiencing mental health issues. Interventions need to target high-risk groups identified in this study, including women, older adults, individuals with a family history of T2DM, people with lower education or unstable employment, and students, particularly those who have completed higher degrees and are actively seeking employment, who are more vulnerable to depression. Collaborative care involving physicians, mental health specialists, and diabetes educators can enhance patient outcomes. Furthermore, long-term research is necessary to clarify causal relationships and evaluate the effectiveness of comprehensive psychosocial support programs.

## Conclusions

In Chennai, patients with T2DM frequently experience overlapping psychiatric conditions such as stress, anxiety, and depression. Beyond traditional risk factors like older age, female sex, financial constraints, and a family history of diabetes, students, particularly those completing advanced studies while facing the uncertainties of employment, emerge as a group susceptible to mental health challenges. These findings highlight the necessity of integrated care models that combine psychological support with routine diabetes management. By addressing both metabolic and mental health needs, such approaches can improve patient well-being and help reduce the overall burden of disease in this community.

## Appendices

**Table 6:** Questionnaire COPD: chronic obstructive pulmonary disease

Section A: socio-demographic details	
(1) Age group	(a) 18-30 years
	(b) 31-40 years
	(c) 41-50 years
	(d) 51-60 years
	(e) Above 60 years
(2) Gender	(a) Male
	(b) Female
(3) Education level	(a) No formal education
	(b) Primary
	(c) Secondary and higher secondary
	(d) Graduate
	(e) Postgraduate and above
(4) Employment status	(a) Employed full-time (working 40+ hours per week)
	(b) Employed part-time (working less than 40 hours per week)
	(c) Unemployed
	(d) Homemaker
	(e) Retired
(5) Monthly household income	(a) Less than ₹10,000
	(b) ₹10,000-24,999
	(c) ₹25,000-50,000
	(d) More than ₹50,000
(6) Type of family	(a) Nuclear family
	(b) Joint
	(c) Extended
(7) Place of residence	(a) Urban
	(b) Rural
Section B: clinical parameters	
(8) Do you have any chronic illnesses other than diabetes?	(a) Yes, hypertension
	(b) Yes, I have cardiovascular disease (heart-related issues)
	(c) Yes, I have a chronic respiratory condition (e.g., asthma, COPD)
	(d) No, I do not have any other illness
(9) Are you currently on any medications other than for type 2 diabetes mellitus?	(a) Yes, I am taking medication for hypertension
	(b) Yes, I am taking medications for heart-related disease
	(c) Yes, I am taking medications for a respiratory condition
	(d) No, I am not taking any other medications
(10) Family history of mental illness	(a) Yes
	(b) No
(11) How often have you consumed alcohol in the past 12 months?	(a) Never
	(b) Less than once a month
	(c) 1-3 times a month
	(d) Daily or almost daily
Depression Anxiety Stress Scale-21 (DASS-21)	
I found it hard to wind down	0 - did not apply to me at all
	1 - applied to me to some degree or some of the time
	2 - applied to me to a considerable degree or a good part of the time
	3 - applied to me very much or most of the time
I was aware of the dryness of my mouth	0 - did not apply to me at all
	1 - applied to me to some degree or some of the time
	2 - applied to me to a considerable degree or a good part of the time
	3 - applied to me very much or most of the time
I couldn't seem to experience any positive feelings at all	0 - did not apply to me at all
	1 - applied to me to some degree or some of the time
	2 - applied to me to a considerable degree or a good part of the time
	3 - applied to me very much or most of the time
I experienced breathing difficulty (e.g., excessively rapid breathing, breathlessness in the absence of physical exertion)	0 - did not apply to me at all
	1 - applied to me to some degree or some of the time
	2 - applied to me to a considerable degree or a good part of the time
	3 - applied to me very much or most of the time
I found it difficult to work up the initiative to do things	0 - did not apply to me at all
	1 - applied to me to some degree or some of the time
	2 - applied to me to a considerable degree or a good part of the time
	3 - applied to me very much or most of the time
I tended to overreact to situations	0 - did not apply to me at all
	1 - applied to me to some degree or some of the time
	2 - applied to me to a considerable degree or a good part of the time
	3 - applied to me very much or most of the time
I experienced trembling (e.g., in the hands)	0 - did not apply to me at all
	1 - applied to me to some degree or some of the time
	2 - applied to me to a considerable degree or a good part of the time
	3 - applied to me very much or most of the time
I felt that I was using a lot of nervous energy	0 - did not apply to me at all
	1 - applied to me to some degree or some of the time
	2 - applied to me to a considerable degree or a good part of the time
	3 - applied to me very much or most of the time
I was worried about situations in which I might panic and make a fool of myself	0 - did not apply to me at all
	1 - applied to me to some degree or some of the time
	2 - applied to me to a considerable degree or a good part of the time
	3 - Applied to me very much or most of the time
I felt that I had nothing to look forward to	0 - did not apply to me at all
	1 - applied to me to some degree or some of the time
	2 - applied to me to a considerable degree or a good part of the time
	3 - applied to me very much or most of the time
I found myself getting agitated	0 - did not apply to me at all
	1 - applied to me to some degree or some of the time
	2 - applied to me to a considerable degree or a good part of the time
	3 - applied to me very much or most of the time
I found it difficult to relax	0 - did not apply to me at all
	1 - applied to me to some degree or some of the time
	2 - applied to me to a considerable degree or a good part of the time
	3 - applied to me very much or most of the time
I felt downhearted and blue	0 - did not apply to me at all
	1 - applied to me to some degree or some of the time
	2 - applied to me to a considerable degree or a good part of the time
	3 - applied to me very much or most of the time
I was intolerant of anything that kept me from getting on with what I was doing	0 - did not apply to me at all
	1 - applied to me to some degree or some of the time
	2 - applied to me to a considerable degree or a good part of the time
	3 - applied to me very much or most of the time
I felt I was close to panic	0 - did not apply to me at all
	1 - applied to me to some degree or some of the time
	2 - applied to me to a considerable degree or a good part of the time
	3 - applied to me very much or most of the time
I was unable to become enthusiastic about anything	0 - did not apply to me at all
	1 - applied to me to some degree or some of the time
	2 - applied to me to a considerable degree or a good part of the time
	3 - applied to me very much or most of the time
I felt I wasn't worth much as a person	0 - did not apply to me at all
	1 - applied to me to some degree or some of the time
	2 - applied to me to a considerable degree or a good part of the time
	3 - applied to me very much or most of the time
I felt that I was rather touchy	0 - did not apply to me at all
	1 - applied to me to some degree or some of the time
	2 - applied to me to a considerable degree or a good part of the time
	3 - applied to me very much or most of the time
I was aware of the action of my heart in the absence of physical exertion (e.g., sense of heart rate increase, heart missing a beat)	0 - did not apply to me at all
	1 - applied to me to some degree or some of the time
	2 - applied to me to a considerable degree or a good part of the time
	3 - Applied to me very much or most of the time
I felt scared without any good reason	0 - did not apply to me at all
	1 - applied to me to some degree or some of the time
	2 - applied to me to a considerable degree or a good part of the time
	3 - applied to me very much or most of the time
I felt that life was meaningless	0 - Did not apply to me at all
	1 - applied to me to some degree or some of the time
	2 - applied to me to a considerable degree or a good part of the time
	3 - applied to me very much or most of the time
